# Dimensionality Reduction and Louvain Agglomerative Hierarchical Clustering for Cluster-Specified Frequent Biomarker Discovery in Single-Cell Sequencing Data

**DOI:** 10.3389/fgene.2022.828479

**Published:** 2022-02-07

**Authors:** Soumita Seth, Saurav Mallik, Tapas Bhadra, Zhongming Zhao

**Affiliations:** ^1^ Department of Computer Science & Engineering, Aliah University, Kolkata, India; ^2^ Center for Precision Health, School of Biomedical Informatics, The University of Texas, Health Science Center at Houston, Houston, TX, United States; ^3^ Human Genetics Center, School of Public Health, The University of Texas Health Science Center at Houston, Houston, TX, United States

**Keywords:** single-cell sequencing data analysis, dimensionality reduction, principal component analysis(PCA), agglomerative hierarchical clustering, modularity optimization, cluster specified biomarkers

## Abstract

The major interest domains of single-cell RNA sequential analysis are identification of existing and novel types of cells, depiction of cells, cell fate prediction, classification of several types of tumor, and investigation of heterogeneity in different cells. Single-cell clustering plays an important role to solve the aforementioned questions of interest. Cluster identification in high dimensional single-cell sequencing data faces some challenges due to its nature. Dimensionality reduction models can solve the problem. Here, we introduce a potential cluster specified frequent biomarkers discovery framework using dimensionality reduction and hierarchical agglomerative clustering Louvain for single-cell RNA sequencing data analysis. First, we pre-filtered the features with fewer number of cells and the cells with fewer number of features. Then we created a Seurat object to store data and analysis together and used quality control metrics to discard low quality or dying cells. Afterwards we applied global-scaling normalization method “LogNormalize” for data normalization. Next, we computed cell-to-cell highly variable features from our dataset. Then, we applied a linear transformation and linear dimensionality reduction technique, Principal Component Analysis (PCA) to project high dimensional data to an optimal low-dimensional space. After identifying fifty “significant”principal components (PCs) based on strong enrichment of low p-value features, we implemented a graph-based clustering algorithm Louvain for the cell clustering of 10 top significant PCs. We applied our model to a single-cell RNA sequential dataset for a rare intestinal cell type in mice (NCBI accession ID:GSE62270, 23,630 features and 1872 samples (cells)). We obtained 10 cell clusters with a maximum modularity of 0.885 1. After detecting the cell clusters, we found 3871 cluster-specific biomarkers using an expression feature extraction statistical tool for single-cell sequencing data, Model-based Analysis of Single-cell Transcriptomics (MAST) with a   log _2_ *FC* threshold of 0.25 and a minimum feature detection of 25*%*. From these cluster-specific biomarkers, we found 1892 most frequent markers, i.e., overlapping biomarkers. We performed degree hub gene network analysis using Cytoscape and reported the five highest degree genes (*Rps4x*, *Rps18*, *Rpl13a*, *Rps12* and *Rpl18a*). Subsequently, we performed KEGG pathway and Gene Ontology enrichment analysis of cluster markers using David 6.8 software tool. In summary, our proposed framework that integrated dimensionality reduction and agglomerative hierarchical clustering provides a robust approach to efficiently discover cluster-specific frequent biomarkers, i.e., overlapping biomarkers from single-cell RNA sequencing data.

## 1 Introduction

Single-cell RNA sequencing (scRNAseq) technology plays a vital role in medical fields such as oncology, digestive and urinary systems, microbiology, neurology, reproduction, and immunology (Tang et al., 2019). For identifying the genome, transcriptome single-cell RNA sequencing technology may be used. Additionally, it can obtain other multi-omics information to disclose the differences in cell populations and evolutionary relationships among cells. However, there are some limitations in traditional sequencing technology. For example, it can only find the average of many cells, and it fails to analyze a few cells. Traditional sequencing technology invokes the probability of losing cellular heterogeneity information, a problem which is overcome by single-cell RNA sequencing technology, since it can detect heterogeneity among individual cells. The workflow of single-cell sequencing involves isolating a single cell from a group of cells, studying cell heterogeneity, molecular mapping, and tracking immune infiltration and epigenetic changes. The ongoing research interests on single-cell sequential analysis includes identification of existing and novel types of cells, depiction of cells, cell fate prediction, classification of several types of tumor, investigation of heterogeneity in different cells (Huh et al., 2020). Single-cell clustering plays a crucial role in conducting such analysis. In single-cell sequencing analysis, cell clustering is required for detection and examination of cluster-specific gene signatures, reconciliation of cell type configuration to mark the gene signature as differentially expressed, and simplification of the bulk RNA-seq expression data by removing noise. Due to its importance, many scRNA-seq clustering methods are available in scientific literature. However, different clustering methods employs distinct strategies to improve the accuracy of clustering results, such as, importing various types of distance metrics, and using different techniques for dimension reduction and calculating number of clusters. Every clustering method has its own strengths as well as its drawbacks. For cell clustering, it is recommended to use two or more clustering techniques to increase accuracy and comprehensive overviews. However, it is critical to select the best clustering method, especially when cluster labels are unknown. In 2020, Huh et al. (2020) provided a mixture model based probabilistic framework for single cell clustering by deploying multiple clustering methods or aggregate clustering like t-SNE + *k*-means with ADPclust, an automated method capable of computing number of clusters and centroids of clusters. The authors claimed that their model has improved clustering performance for labeling individual single-cells, as well as the accurate estimation of number of clusters. However, their method faces several analytical and technical challenges in the analysis of large-scale single cell data due to high dimensionality, sparse matrix computation, and rare cell detection (Feng et al., 2020). Specifically, the high dimensionality and sparse matrix creates the curse of dimensionality. As a result, several techniques like quality control, mapping, quantification, dimensionality reduction, clustering, finding trajectories, and identifying differentially expressed genes etc. needs to be included for the computational analysis of scRNA-seq data. The two most important techniques among these are dimensionality reduction and clustering, which play effective roles on downstream analysis.

Cluster identification in high dimensional single-cell sequencing data struggles with high dimensionality. To solve this problem and other undesirable properties of high-dimensional space, dimensionality reduction models in various research fields are needed. Unsupervised dimensionality reduction methods are efficient to discover natural grouping of a set of samples in high-dimensional feature space. The k-means algorithm, a renowned widely clustering algorithm in data mining (Wu et al., 2008), is used in the Monocle scRNA-seq toolkit (Qiu et al., 2017). BackSPIN (Zeisel et al., 2015) and pcaReduce (Zurauskiene and Yau, 2016) are an extension of hierarchical clustering by importing the mechanism of dimension reduction after each split or merge. This procedure improves the accuracy of small size cluster identification. Two main categories of dimensionality reduction are feature selection and extraction. Feature selection involves selecting a subset of features from the original dataset. Feature extraction derives information from the original set of features and builds a new subspace of features. Principal Component Analysis (PCA) is a commonly used algorithm for unsupervised feature extraction. PCA is normally applied on linear models which map high-dimensional data to low dimensional space (Bartenhagen et al., 2010).

In the last 2 decades, dimensionality reduction and clustering has gathered increasing research interest for single-cell RNA sequencing data analysis. In this article, we provide a dimensionality reduction integrated clustering model for detecting cluster-specific biomarkers in single-cell sequencing data. We applied it in a single-cell RNA sequential dataset for a rare intestinal cell type in mice (NCBI accession ID:GSE62270) (Grün et al., 2015). We pre-filtered the features with fewer number of cells and the cells with fewer number of features. After that, we create a Seurat object to store data and analysis together for the dataset. Then we use quality control metrics for discarding low quality or dying cells. Subsequently, we applied global-scaling normalization method “LogNormalize” for data normalization. Next, we compute cell-to-cell highly variable features from our dataset and performed a linear transformation and linear dimensionality reduction technique, PCA to project high dimensional data to an optimal low-dimensional space. After identifying fifty “significant” principal components (PCs) based on strong enrichment of low p-value features, we implemented a graph-based clustering on the cell of top 10 “significant” PCs using the modularity optimization agglomerative clustering algorithm, Louvain. After detecting the cell clusters, we identified cluster-specific biomarkers using an expression feature extraction statistical tool for single-cell sequencing data, Model-based Analysis of Single-cell Transcriptomics (MAST). We further performed degree hub gene network analysis using *Cytoscape* and found the five top degree markers (*Rps4x*, *Rps18*, *Rpl13a*, *Rps12* and *Rpl18a*). After that, we performed Gene Set Enrichment Analysis (GESA) to determine enriched KEGG pathways and Gene Ontology (GO) terms including Biological Process (BP), Cellular Component (CC), and Molecular Function (MF) on the set of clusters specified markers using David 6.8 software tool (Dennis et al., 2003). In summary, our proposed integrated framework using dimensionality reduction and hierarchical agglomerative clustering efficiently discovers cluster-specific frequent biomarkers, i.e. overlapping biomarkers from single-cell RNA sequencing data.

## 2 Materials and Methods

**FIGURE 1:**
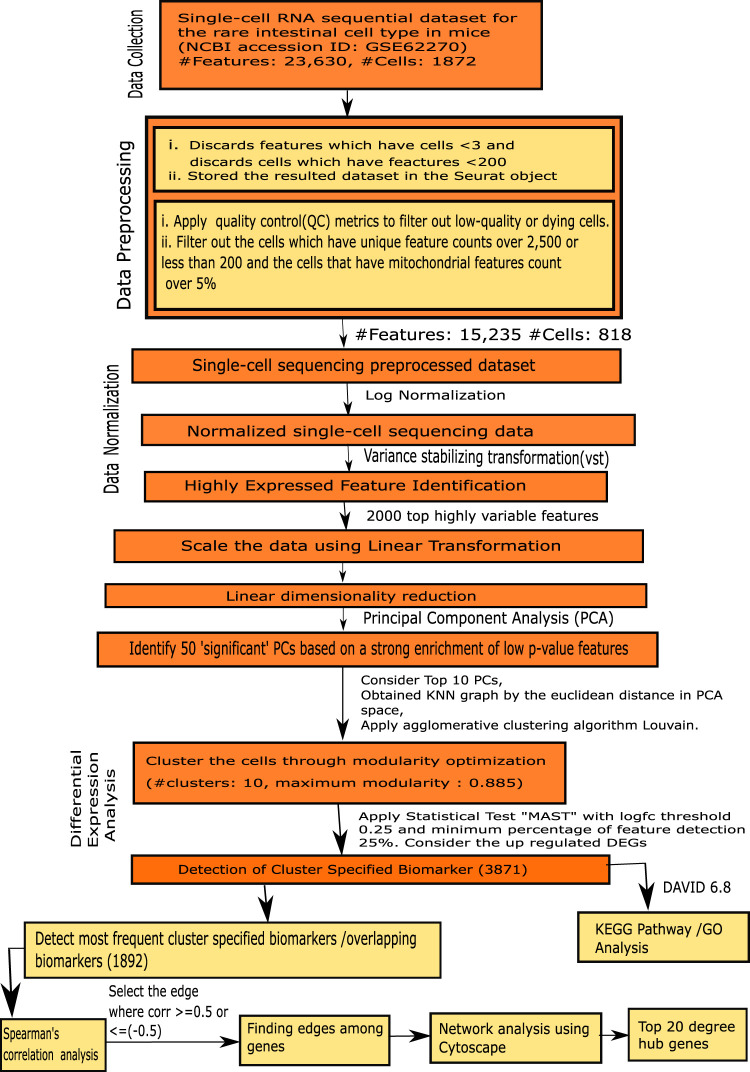
Flowchart of the proposed framework.

The steps of our proposed framework are demonstrated as follow, as well as in Figure 1.

### 2.1 Data Collection

In this study, we used a single-cell RNA sequential dataset for the rare intestinal cell type in mice (NCBI accession ID: GSE62270) which has 23,630 features and 1872 samples (cells) (Grün et al., 2015).

### 2.2 Preprocessing of Single-Cell RNA Sequencing Data

In this article, we provided an extensive analysis by integrating dimensionality reduction technique and clustering algorithm for detecting cluster-specific frequent biomarkers in single-cell RNA sequencing (scRNAseq) data. In the following subsections, we describe procedures to preprocess a scRNA-seq dataset.

#### 2.2.1 Data Preprocessing

Data preprocessing is an important step for further analysis. First, we discarded features and cells that do not have minimum number of cells and features respectively. Afterwards, we created a Seurat object to store our data matrix, allowing us to store both data (like input feature sample matrix) and analysis (like PCA, or clustering results) together for a single-cell dataset (Butler et al., 2018; Stuart et al., 2019a).

#### 2.2.2 Compute Quality Control Metrics and Cell Filtration

In this step, we explored QC metrics based on user defined criteria for the selection and filtration of cells. We first filtered out empty cells. Filtering low-quality or dying cells is an important preprocessing strategy for scRNAseq data (Ilicic et al., 2016). Generally, the cells having a few genes is considered low-quality cells, or dying cells. Choosing appropriate thresholds to keep high quality cells without removing biologically relevant cell types is an important factor. We defined a threshold range (200–2,500) for a number of unique features in a cell and filtered out the cells that does not meet the criteria. To avoid removing biologically relevant cell types, we computed mitochondrial QC metrics to calculate the mitochondrial count percentage from the set of features. We also defined an upper bound threshold for the percentage of mitochondrial count (5*%*) and filtered out the cells above the upper bound.

#### 2.2.3 Data Normalization

After the cell filtration, data normalization was performed using the global-scaling normalization method “LogNormalize” which divides the specific feature counts of each cell by the total counts of that cell and multiplies it by a scaling factor (10^4^) and then performs natural log-transformation. In scRNAseq context, a Z-scoring metric indicates how much the frequency of one cell for a given feature deviates from the mean of the frequencies of all cells for that feature. It is Z-scores are calculated from the log-normalized counts. Suppose, the transcription value *Tr*_*ij*_ where *i* = feature (gene) and *j* = cell. Let, *N*_*j*_ be the total counts for the cell *j*. We can formulate this normalization procedure by *log*_10_{(*Tr*_*ij*_/*N*_*j*_)∗10^4^} for each feature *i* in respect to each cell *j*.

### 2.3 Highly Variable Features Identification

Next, we computed cell-to-cell highly variant features from our dataset. The feature which are highly expressed in some cells and lowly expressed in others cells is noted as “highly variable” features. Such highly variable genes play an important role in downstream analysis in single-cell datasets through highlighting the biological signal (Brennecke et al., 2013). In this work, we used a mean-variance relationship model, i.e., variance stabilizing transformation (vst) to identify highly variable features (Stuart et al., 2019b). Mean-variance relationship is inherent to scRNA-seq. To determine this relationship from the data, first we evaluated the means and variances of each feature. Afterwards, to predict the variance of each feature as a function of its mean, we fixed a curve and calculated a local fitting of polynomials of degree 2. The global fit is defined by a regularized estimator of variance, where the mean of a feature is already given. This may be used for standardizing feature count, to prevent discarding higher-than-expected variations. Since the expected variance is already given, we define the transformation as,

$$y_{ij}=\frac{p_{ij}-{\bar{p}}_{i}}{\sigma_{i}}$$ (1)

where, *y*_*ij*_ is the standardized value of feature *i* in cell *j* and *p*_*ij*_ denotes the raw value of feature *i* in cell *j*, *p*_*i*_ is the mean value of feature *i* and *σ*_*i*_ is the expected standard deviation of feature *i* derived from the global mean-variance fit. In order to decrease the effect of technical outliers, we fixed the maximum standardized value as $\sqrt{M}$ , where *M* is the total number of cells. The variances of standardized values across all cells are computed for each feature. This variance constitutes a measure of single cell dispersion after controlling for mean expression, which helps us in ranking features. After ranking, we choose the 2,000 top features which have the highest standardized variance as “highly variable” features.

### 2.4 Linear Transformation and Linear Dimensionality Reduction

Linear dimensionality reduction is a keystone step for downstream analysis of high dimensional data (Cunningham and Ghahramani, 2015). Linear dimensionality reduction methods accept high dimensional data as input and project them to an optimal low-dimensional space. There are various methods that capture several feature interests like covariance, dynamical structure, correlation between data sets, input-output relationships, and margin between data classes etc. Feature selection and feature extraction is a part of linear dimensionality reduction. We used the well-known linear dimensionality reduction technique, PCA which captures covariance as feature interest. PCA selects and extracts data based on increasing variance. The features with the maximum variance are marked as “Principal Component”. Before applying PCA, we performed a linear transformation to standardize the data. As a result, weight was equally distributed which prevents the highly-expressed features from being dominant. Then we performed PCA technique on the scaled data and considered the computed 2000 “highly variable” features as a feature subset. PCA technique maximized interpretability and minimized information loss simultaneously (Jolliffe and Cadima, 2016). To determine the dimensionality of a dataset, we implemented a resampling test through JackStraw procedure through which we obtained a subset of the data. A random permutation (taken 1*%* as default) and PCA were conducted on the subset. To construct a null distribution of feature scores, we rerun the PCA technique and repeats the procedure. Here, we identified fifty ‘significant’ principal components (PCs) based on a strong enrichment of low p-value features (Macosko et al., 2015).

### 2.5 Cell Cluster

In this step, we applied graph-based clustering on our data and considered the first 10 PCs for cluster analysis. First, we have obtained a K-nearest neighbor (KNN) graph by the Euclidean distance in PCA space. We calculated the edge weight between two cells through the Jaccard similarity which is defined by shared overlap between cells. Suppose, there are two cells C1 and C2, Jaccard similarity is defined by

$$J\left(C1,C2\right)=\frac{\left|C1\cap C2\right|}{\left|C1\cup C2\right|}\times 100$$ (2)

Cells with maximum similar features have high Jaccard similarity percentage.

Next, we applied modularity optimization agglomerative clustering technique, Louvain algorithm (Blondel et al., 2008), for cell clustering. Modularity is a strong QC step for community detection (clustering), invented by M.E.J Newman in 2006 (Newman, 2006). The modularity of a graph partition is measured by the comparison between the number of interactions inside the clusters and the number of interactions between clusters. The modularity value lies within the range [-1,1]. The value of modularity (Q) is formulated by

$$Q=\frac{1}{2e}\underset{i,j}{\sum }\left[Adj_{ij}-\frac{w_{i}w_{j}}{2e}\right]\delta \left(Cl_{i},Cl_{j}\right)$$ (3)

where, *Adj*_*ij*_ denotes weight of the edge between *i* and *j* of our KNN graph, i.e. adjacency matrix, *w*_*i*_ = *∑*_*j*_ *Adj*_*ij*_ is the total weights of the edges attached to vertex (here, cell) *i*, *Cl*_*i*_ is the cluster to which cell *i* is assigned, *δ*-function is derived as *δ*(*x*.*y*) = 1 when *x* = *y*, otherwise it is 0 and $e=\frac{1}{2}\sum_{ij}Adj_{ij}$ (Blondel et al., 2008).

The objective of modularity optimization is to maximize the average modularity of computed clusters. Blondel et al. (2008) (Blondel et al., 2008) developed a modularity optimization algorithm, Louvain Algorithm. The prime workflow of this method is executed in two phases, which are repeated iteratively (Kirianovskii et al., 2016). In phase 1, they maximized the local modularity by moving each node to neighbor’s communities. In details, for each node *i*, authors found the neighbors *j* of *i* and evaluated the modularity gain of removing *i* from its assigned community and by assigning it in the community (cluster) of *j* (*Cl*_*j*_). The node *i* is placed in the community (cluster) which gives maximum modularity gain. Modularity gain should be positive. The Modularity gain Δ*Q* is computed by

$$\begin{array}{l} \Delta Q=\left[\frac{\underset{Cl_{j},Cl_{j}}{\sum }+\underset{i,Cl_{j}}{\sum }}{2e}-{\left(\frac{\underset{Cl_{j}}{\sum }+\underset{i}{\sum }}{2e}\right)}^{2}\right]\\ -\left[\frac{\underset{Cl_{j},Cl_{j}}{\sum }}{2e}-{\left(\frac{\underset{Cl_{j}}{\sum }}{2e}\right)}^{2}-{\left(\frac{\underset{i}{\sum }}{2e}\right)}^{2}\right] \end{array}$$ (4)

where, $\sum_{Cl_{j},Cl_{j}}$ denotes the weights sum of the links which lie in *Cl*_*j*_, $\sum_{i,Cl_{j}}$ refers the weights sum of the links from node *i* to nodes in *Cl*_*j*_, $\sum_{Cl_{j}}$ denotes the sum of the weights of the links incident to nodes in *Cl*_*j*_, *∑*_*i*_ refers the weights sum of the links incident to node *i* and *e* denotes the sum of the weights of all the links in the graph.

When the modularity gain reaches local maximum, it is proceeded to the next phase. In the second phase, authors built a new network by assigning communities found in the first phase as nodes and incorporating the fact that the weights of the links between new nodes are nothing but weight sum of the links between nodes of corresponding two communities. The Links between nodes of a same community, are considered the self-loops in the new network. Two steps are repeated until there are no more variation in modularity gain and modularity maximum is retained. We applied the community detection algorithm in our work for clustering the cells by using R tool FindCluster () with the parameter “resolution” for setting the granularity of downstream analysis, which controls the number of clusters. Increasing the value of resolution parameter, more clusters were found. According to the benchmark, fixing this parameter to the range 0.4–1.2 typically returns significant results for single-cell datasets containing around 3,000 cells. We also applied non-linear dimensional reduction technique, Uniform Manifold Approximation and Projection (UMAP), to visualize the similar cells of graph-based clusters in low-dimensional space by considering the same number of PCs we found during cluster analysis (Myasnikov, 2020).

### 2.6 Finding the Cluster-Specific Biomarkers

We describe how we found differentially expressed genes from each cluster, i.e., detecting cluster specified biomarkers. For detecting differentially expressed features (markers) we applied commonly used distinct expression feature extraction statistical tool for single-cell sequencing data, Model-based Analysis of Single-cell Transcriptomics (MAST) (Finak et al., 2015). We considered each cluster as one group (Experimental Group) and all rest clusters as another group (Control Group) and repeated the procedure for all clusters to find differentially expressed genes from each cluster. MAST modeled the gene expression matrix by a two-part generalized regression model. To model the gene expression rate, it developed logistic regression and to apply condition on a cell expressing the gene, it used Gaussian distribution model at the expression level (Finak et al., 2015). MAST model is highly applicable in bimodal expression distributions where expression is either strongly non-zero or non-detectable (Dal Molin et al., 2017). MAST model is developed by (Finak et al., 2015). The authors denoted *Y* = [*Y*_*ig*_] as the rate of expression and the level of expression for an independent gene *g* and cell *i*. They used an indicator *Z* = [*z*_*ig*_] to indicate whether the gene *g* is expressed in the cell *i* or not (i.e., *z*_*ig*_ = 0 if *y*_*ig*_ = 0 and *z*_*ig*_ = 1 if *y*_*ig*_ > 0). The Authors formulated a logistic regression model for the discrete variable *Z* and a Gaussian linear model for the continuous variable (*Y*|*Z* = 1) as follow (Finak et al., 2015; Dal Molin et al., 2017):

$$logit\left(P_{r}\left(Z_{ig}=1\right)\right)=X_{i}\beta_{g}^{D}$$ (5)

$$P_{r}=\left(Y_{ig}=y|Z_{ig}=1\right)=N\left(X_{i}\beta_{g}^{C},\sigma_{g}^{2}\right)$$ (6)

*X*_*i*_ is the design matrix. Fraction of genes being expressed and detectable in each cell, is termed as cellular detection rate (CDR). The CDR for cell *i* is formulated as:

$$CDR_{i}=\frac{1}{N}\overset{N}{\underset{g=1}{\sum }}z_{ig}$$ (7)

CDR variability is modelled by a covariate variable (a column in the design matrix *X*_*i*_), in the discrete and continuous models. CDR covariate is important because the discrete analog of global normalization, which can detect genuine gene co-expression by decreasing background correlation between features. *N* is the total number of genes in a cell. The parameters of this model are fitted by an empirical Bayesian framework that improves the inference for genes with sparse expression. Likelihood ratio test is used for testing differential expression. *β* is the likelihood estimator, *σ* denotes variance.

In our method, besides MAST model, we set the logarithm of fold change with base 2 (log _2_ *FC*) threshold (which measure how much a feature to be differentially expressed) to 0.25 and considered the up-regulated differentially expressed genes of all clusters as markers. Simultaneously, we set another parameter min.pct to 0.25, which holds a minimum percentage of a feature detection in either of the two groups, Control group and Experimental group, (i.e., 25*%*). We detected cluster-specific biomarkers based on p-value and Bonferroni corrected adjusted p-value, which is called as False Discovery Rate (FDR). Our main goal was to find frequent cluster markers, which are overlapping markers.

### 2.7 Hub Gene Finding

In the next step, we applied Spearman’s correlation analysis on the cluster-specific and most frequent biomarkers identified by our method. This step aims to obtain the active edges among genes having correlation value ≥ 0.5 or ≤ − 0.5. After obtaining the set of active edges, we performed degree centrality analysis through *Cytoscape* online tool (Shannon et al., 2003) and determined degree scores for each marker. We marked top 20 markers (hubs) by degree.

### 2.8 Gene Set Enrichment Analysis

Gene Set Enrichment Analysis (GESA) is used to assess the potential function, biological significance, and disease relevance of a list of signature genes. After detecting cluster-specific biomarkers and differentially expressed genes, we used KEGG pathways and Gene Ontology (GO) annotations (three domains: Biological Process (BP), Cellular Component (CC), and Molecular Function (MF)) by DAVID 6.8 software (Dennis et al., 2003). We obtained all KEGG pathways and Gene Ontology (GO) terms along with the number of genes in that pathway or GO-term, enriched adjusted p-value and FDR. We kept KEGG pathways or GO terms whose FDR were less than or equal to 0.05.

## 3 Result and Discussion

**FIGURE 2:**
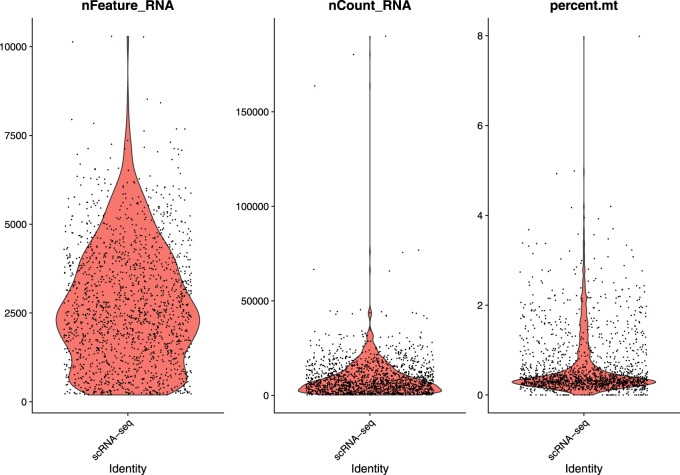
Visualize QC metrics as a violin plot.

**FIGURE 3:**
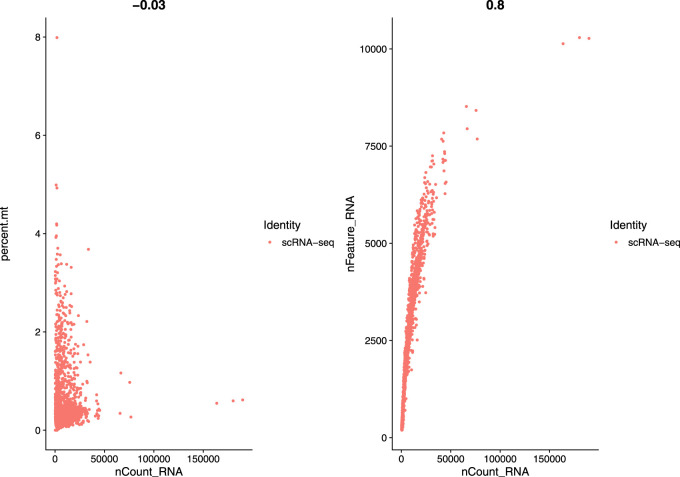
FeatureScatter plot to visualize feature-feature relationships.

In this study, we used a single-cell RNA sequential dataset for the rare intestinal cell type in mice (GEO ID: GSE62270) which has 23,630 features and 1872 samples. We preprocessed our scRNA-seq data for further analysis. We first filtered out features that have less than three cells and cells that have less than 200 features and stored the resulted dataset as a Seurat object. The resulted dataset contained 15,235 features and 1644 samples. We followed some quality control (QC) metrics to filter out low-quality or dying cells which create mitochondrial pollution. We also calculated QC metrics with PercentageFeatureSet function, a function that can compute the percentage of mitochondrial feature count from a set of all types of features. Here we considered a set of all genes starting with “MT” as the set of mitochondrial genes. We filtered out the cells which have unique feature counts over 2,500 or less than 200 and the cells that have mitochondrial features count over 5*%*. After these preprocessing steps, our dataset contained 818 cells (samples) with 15,235 features. Figure 2 visualizes QC metrics as a violin plot and Figure 3 displays the visualization of feature-feature relationships.

**FIGURE 4:**
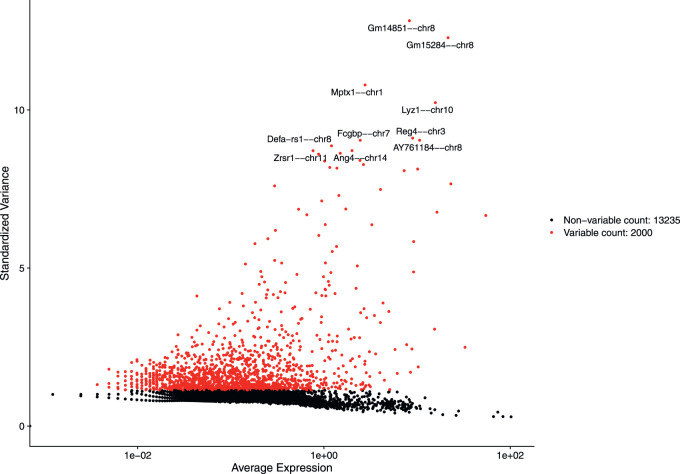
Variable features with labels.

After discarding unwanted cells, we applied log normalization method to normalize our dataset and applied feature selection method “vst” to identify highly variable features. The 2,000 highly variable features to conduct further downstream analysis were obtained. Figure 4 shows 2,000 highly variable features with some feature labels.

**FIGURE 5:**
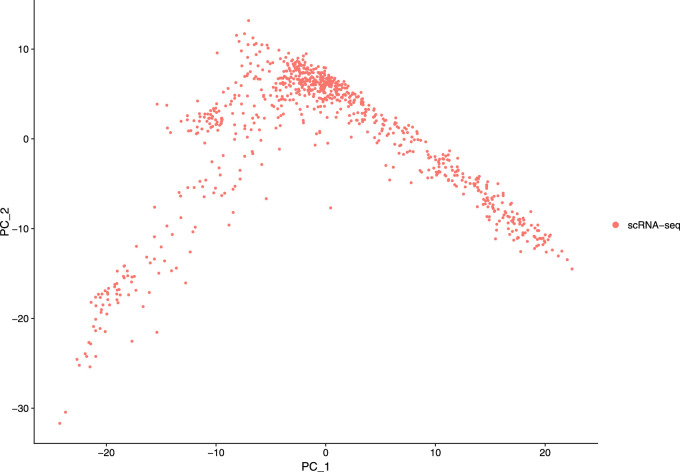
DimPlot of two principal components (PC1 Vs PC2).

**FIGURE 6:**
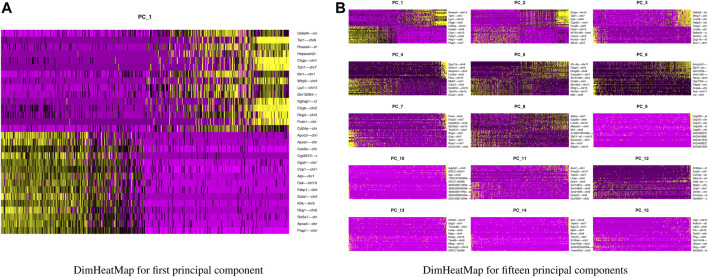
DimHeatMap for principal components. **(A)** DimHeatMap for first principal component. **(B)** DimHeatMap for fifteen principal components.

**FIGURE 7:**
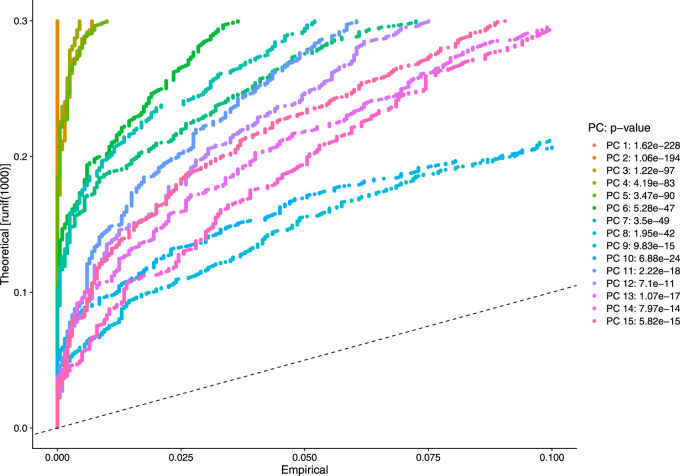
Visualization of strong enrichment of features with low p-values.

Our next step is to perform PCA, a linear dimensional reduction technique, where we used 2000 highly variable features as input. First, we applied linear transformation as standard pre-processing step of dimensional reduction technique. Through linear transformation, equal weights are assigned in downstream analysis, and the possibility of domination by highly expressed features is diminished. Then we performed PCA on the scaled data. We obtained fifty principal components (PC) with several positive and negative features. Supplementary Figure S1 shows the dimension reduction plot for two components, and Figure 5 refers to Dim Plot of first principal component *vs*. second principal component. DimHeatMap for principal components is shown in Figure 6 (Figure 6A; Figure 6B represent DimHeatMap for PC1 and for PC1-PC15 respectively). The PCs which have strong enrichment of features with low p-values are denoted as “Significant” PCs. Figure 7 shows fifteen “Significant” PCs with their respective p-values.

**FIGURE 8:**
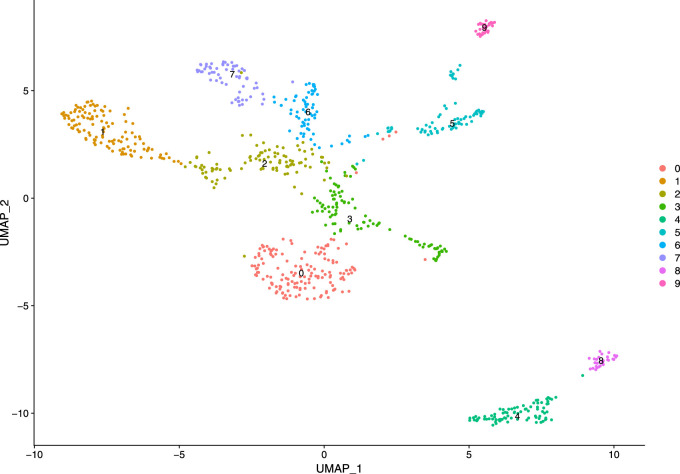
Visualization of clusters.

Subsequently, we constructed a K-nearest neighbor (KNN) graph based on the Euclidean distance in PCA space and computed the edge weights between any two cells through the Jaccard similarity. We considered first 10 PCs as the dimensionality of the dataset. Our KNN graph contained 818 nodes and 20,511 edges. For cell clustering, we applied a modularity optimization technique (Louvain algorithm). This clustering function uses a parameter, resolution, for setting “granularity” of the downstream clustering. If we increase the value of resolution parameter, it leads to a greater number of clusters. According to benchmark, fixing this parameter to the range 0.4–1.2 typically returns significant results for single-cell datasets containing around 3000 cells. For our analysis, the parameter is set as 0.5, and we obtained 10 clusters with maximum modularity 0.885 1. After cell clustering, we used a non-linear dimensional reduction technique, UMAP, to visualize the similar cells of graph-based clusters in low-dimensional space (referred Figure 8). We considered the same PCs which were found in the cluster analysis.

Our final step is finding cluster specified biomarkers, i.e., differentially expressed features. For every cluster, we considered the cluster as one group and rest clusters as another group. To identify differentially expressed features between two groups of cells, we applied the statistical test “MAST”. “MAST” uses a hurdle model tailored to scRNA-seq data. In this procedure, we set the log _2_ *FC* threshold, measuring how much a feature is differentially expressed, to 0.25 and considered the up-regulated differentially expressed genes of all clusters as markers. Another parameter, min.pct which holds a minimum percentage of a feature detection in either of the two groups, is set to 0.25. We identified 6394 cluster-specific markers with their respective clusters of which 3871 markers are unique. There were some overlapping markers lying in more than one cluster. These markers are termed as “frequent marker”. We observed 1892 frequent biomarkers in our analysis. Thirty top frequent biomarkers are presented in Table 1, accompanied by frequency and corresponding adjusted p-values based on Bonferroni correction using all genes in the dataset. This adjusted p-values also termed as false discovery rate (FDR) adjusted p-value. We provided the list of all frequent biomarkers in a Supplementary Table S2.

**TABLE 1:** Top 30 cluster specified frequent biomarkers.

Marker name^a^	Frequency	Specified clusters	FDR
*Atp*5*j*2	$\frac{5}{10}$	Group1: Cluster0 *vs.* Group2: Rest all clusters	1.82 × 10^–06^
		Group1: Cluster1 *vs.* Group2: Rest all clusters	7.12 × 10^–03^
		Group1: Cluster3 *vs.* Group2: Rest all clusters	2.49, ×, 10^–07^
		Group1: Cluster6 *vs.* Group2: Rest all clusters	1.00, ×, 10^–00^
		Group1: Cluster7 *vs.* Group2: Rest all clusters	1.00, ×, 10^–00^
*ERCC*_00009	$\frac{5}{10}$	Group1: Cluster2 *vs.* Group2: Rest all clusters	8.69, ×, 10^–28^
		Group1: Cluster3 *vs.* Group2: Rest all clusters	2.08 × 10^–02^
		Group1: Cluster6 *vs.* Group2: Rest all clusters	4.82 × 10^–05^
		Group1: Cluster7 *vs.* Group2: Rest all clusters	3.24 × 10^–01^
		Group1: Cluster8 *vs.* Group2: Rest all clusters	1.14 × 10^–04^
*Nedd*4	$\frac{5}{10}$	Group1: Cluster0 *vs.* Group2: Rest all clusters	1.07 × 10^–8^
		Group1: Cluster3 *vs.* Group2: Rest all clusters	3.45 × 10^–07^
		Group1: Cluster4 *vs.* Group2: Rest all clusters	2.29, ×, 10^–04^
		Group1: Cluster5 *vs.* Group2: Rest all clusters	6.97 × 10^–01^
		Group1: Cluster9 *vs.* Group2: Rest all clusters	4.97 × 10^–03^
*Abhd*17*a*	$\frac{4}{10}$	Group1: Cluster1 *vs.* Group2: Rest all clusters	6.52 × 10^–02^
		Group1: Cluster2 *vs.* Group2: Rest all clusters	4.45 × 10^–05^
		Group1: Cluster6 *vs.* Group2: Rest all clusters	1.00, ×, 10^–00^
		Group1: Cluster7 *vs.* Group2: Rest all clusters	8.31 × 10^–02^
*Actn*1	$\frac{4}{10}$	Group1: Cluster0 *vs.* Group2: Rest all clusters	1.29, ×, 10^–02^
		Group1: Cluster4 *vs.* Group2: Rest all clusters	1.50, ×, 10^–06^
		Group1: Cluster5 *vs.* Group2: Rest all clusters	8.56 × 10^–05^
		Group1: Cluster9 *vs.* Group2: Rest all clusters	8.12 × 10^–05^
*Arpc*1*b*	$\frac{4}{10}$	Group1: Cluster0 *vs.* Group2: Rest all clusters	1.04 × 10^–05^
		Group1: Cluster1 *vs.* Group2: Rest all clusters	3.00, ×, 10^–04^
		Group1: Cluster3 *vs.* Group2: Rest all clusters	1.69, ×, 10^–05^
		Group1: Cluster8 *vs.* Group2: Rest all clusters	4.63 × 10^–03^
*Atp*5*b*	$\frac{4}{10}$	Group1: Cluster0 Vs Group2: Rest all clusters	7.59, ×, 10^–11^
		Group1: Cluster3 *vs.* Group2: Rest all clusters	2.34 × 10^–05^
		Group1: Cluster6 *vs.* Group2: Rest all clusters	9.96 × 10^–05^
		Group1: Cluster7 *vs.* Group2: Rest all clusters	1.00, ×, 10^–04^
*B*2*m*	$\frac{4}{10}$	Group1: Cluster0 *vs.* Group2: Rest all clusters	6.49, ×, 10^–15^
		Group1: Cluster1 *vs.* Group2: Rest all clusters	1.14 × 10^–15^
		Group1: Cluster3 *vs.* Group2: Rest all clusters	8.88 × 10^–11^
		Group1: Cluster9 Vs Group2: Rest all clusters	5.56 × 10^–03^
*B*4*galnt*2	$\frac{4}{10}$	Group1: Cluster0 *vs.* Group2: Rest all clusters	9.46 × 10^–16^
		Group1: Cluster1 *vs.* Group2: Rest all clusters	9.37 × 10^–04^
		Group1: Cluster3 *vs.* Group2: Rest all clusters	1.41 × 10^–02^
		Group1: Cluster4 *vs.* Group2: Rest all clusters	5.64 × 10^–07^
*Calm*1	$\frac{4}{10}$	Group1: Cluster1 *vs.* Group2: Rest all clusters	6.53 × 10^–18^
		Group1: Cluster3 *vs.* Group2: Rest all clusters	8.54 × 10^–04^
		Group1: Cluster5 *vs.* Group2: Rest all clusters	1.28 × 10^–05^
		Group1: Cluster9 *vs.* Group2: Rest all clusters	1.65 × 10^–01^
*Cox*5*b*	$\frac{4}{10}$	Group1: Cluster0 *vs.* Group2: Rest all clusters	1.72 × 10^–14^
		Group1: Cluster3 *vs.* Group2: Rest all clusters	9.78 × 10^–07^
		Group1: Cluster6 *vs.* Group2: Rest all clusters	5.75 × 10^–01^
		Group1: Cluster7 *vs.* Group2: Rest all clusters	1.18 × 10^–03^
*Dhcr*24	$\frac{4}{10}$	Group1: Cluster1 *vs.* Group2: Rest all clusters	3.35 × 10^–10^
		Group1: Cluster6 *vs.* Group2: Rest all clusters	3.83 × 10^–03^
		Group1: Cluster7 *vs.* Group2: Rest all clusters	1.15 × 10^–04^
		Group1: Cluster8 *vs.* Group2: Rest all clusters	4.44 × 10^–01^
*Dpysl*2	$\frac{4}{10}$	Group1: Cluster4 *vs.* Group2: Rest all clusters	3.26 × 10^–05^
		Group1: Cluster5 *vs.* Group2: Rest all clusters	2.29, ×, 10^–05^
		Group1: Cluster8 *vs.* Group2: Rest all clusters	8.48 × 10^–08^
		Group1: Cluster9 *vs.* Group2: Rest all clusters	6.19, ×, 10^–01^
*Dst*	$\frac{4}{10}$	Group1: Cluster1 *vs.* Group2: Rest all clusters	1.34 × 10^–06^
		Group1: Cluster5 *vs.* Group2: Rest all clusters	8.44 × 10^–03^
		Group1: Cluster8 *vs.* Group2: Rest all clusters	4.38 × 10^–03^
		Group1: Cluster9 *vs.* Group2: Rest all clusters	3.29, ×, 10^–02^
*Eef*1*a*1	$\frac{4}{10}$	Group1: Cluster0 *vs.* Group2: Rest all clusters	2.84 × 10^–36^
		Group1: Cluster4 *vs.* Group2: Rest all clusters	1.85 × 10^–03^
		Group1: Cluster5 *vs.* Group2: Rest all clusters	4.43 × 10^–02^
		Group1: Cluster9 *vs.* Group2: Rest all clusters	6.96 × 10^–01^
*ERCC*_00003	$\frac{4}{10}$	Group1: Cluster1 *vs.* Group2: Rest all clusters	8.53 × 10^–07^
		Group1: Cluster2 *vs.* Group2: Rest all clusters	6.58 × 10^–10^
		Group1: Cluster3 *vs.* Group2: Rest all clusters	3.11 × 10^–07^
		Group1: Cluster8 *vs.* Group2: Rest all clusters	4.55 × 10^–19^
*ERCC*_00043	$\frac{4}{10}$	Group1: Cluster1 *vs.* Group2: Rest all clusters	3.14 × 10^–09^
		Group1: Cluster2 *vs.* Group2: Rest all clusters	4.50, ×, 10^–10^
		Group1: Cluster3 *vs.* Group2: Rest all clusters	5.90, ×, 10^–03^
		Group1: Cluster8 *vs.* Group2: Rest all clusters	2.37 × 10^–27^
*ERCC*_0007	$\frac{4}{10}$	Group1: Cluster1 *vs.* Group2: Rest all clusters	1.04 × 10^–06^
		Group1: Cluster2 *vs.* Group2: Rest all clusters	7.88 × 10^–02^
		Group1: Cluster3 *vs.* Group2: Rest all clusters	9.58 × 10^–12^
		Group1: Cluster8 *vs.* Group2: Rest all clusters	6.17 × 10^–18^
*Fdps*	$\frac{4}{10}$	Group1: Cluster1 *vs.* Group2: Rest all clusters	4.45 × 10^–04^
		Group1: Cluster2 *vs.* Group2: Rest all clusters	6.28 × 10^–01^
		Group1: Cluster6 *vs.* Group2: Rest all clusters	1.91 × 10^–08^
		Group1: Cluster7 *vs.* Group2: Rest all clusters	8.40, ×, 10^–05^
*Glud*1	$\frac{4}{10}$	Group1: Cluster0 *vs.* Group2: Rest all clusters	9.60, ×, 10^–05^
		Group1: Cluster3 *vs.* Group2: Rest all clusters	3.21 × 10^–01^
		Group1: Cluster5 *vs.* Group2: Rest all clusters	2.70, ×, 10^–11^
		Group1: Cluster9 *vs.* Group2: Rest all clusters	2.21 × 10^–07^
*Hsp*90*b*1	$\frac{4}{10}$	Group1: Cluster0 *vs.* Group2: Rest all clusters	2.48 × 10^–03^
		Group1: Cluster4 *vs.* Group2: Rest all clusters	8.20, ×, 10^–04^
		Group1: Cluster5 *vs.* Group2: Rest all clusters	7.20, ×, 10^–01^
		Group1: Cluster9 *vs.* Group2: Rest all clusters	3.15 × 10^–06^
*Malat*1	$\frac{4}{10}$	Group1: Cluster1 *vs.* Group2: Rest all clusters	2.38 × 10^–12^
		Group1: Cluster2 *vs.* Group2: Rest all clusters	1.91 × 10^–02^
		Group1: Cluster5 *vs.* Group2: Rest all clusters	2.98 × 10^–06^
		Group1: Cluster9 *vs.* Group2: Rest all clusters	2.76 × 10^–07^
*Rpl*17	$\frac{4}{10}$	Group1: Cluster0 *vs.* Group2: Rest all clusters	1.34 × 10^–39^
		Group1: Cluster3 *vs.* Group2: Rest all clusters	1.00, ×, 10^–00^
		Group1: Cluster4 *vs.* Group2: Rest all clusters	1.16 × 10^–02^
		Group1: Cluster9 *vs.* Group2: Rest all clusters	8.70, ×, 10^–01^
*Rpl*4	$\frac{4}{10}$	Group1: Cluster0 *vs.* Group2: Rest all clusters	3.65 × 10^–35^
		Group1: Cluster3 *vs.* Group2: Rest all clusters	7.05 × 10^–02^
		Group1: Cluster4 *vs.* Group2: Rest all clusters	9.36 × 10^–03^
		Group1: Cluster9 *vs.* Group2: Rest all clusters	1.00, ×, 10^–00^
*Rpl*7	$\frac{4}{10}$	Group1: Cluster0 *vs.* Group2: Rest all clusters	5.36 × 10^–29^
		Group1: Cluster4 *vs.* Group2: Rest all clusters	3.64 × 10^–01^
		Group1: Cluster6 *vs.* Group2: Rest all clusters	3.68 × 10^–01^
		Group1: Cluster9 *vs.* Group2: Rest all clusters	1.00, ×, 10^–00^
*Rps*6	$\frac{4}{10}$	Group1: Cluster0 *vs.* Group2: Rest all clusters	1.39, ×, 10^–29^
		Group1: Cluster4 *vs.* Group2: Rest all clusters	5.02 × 10^–03^
		Group1: Cluster6 *vs.* Group2: Rest all clusters	1.00, ×, 10^–00^
		Group1: Cluster9 *vs.* Group2: Rest all clusters	3.52 × 10^–01^
*Sox*9	$\frac{4}{10}$	Group1: Cluster0 Vs Group2: Rest all clusters	3.56 × 10^–06^
		Group1: Cluster4 *vs.* Group2: Rest all clusters	1.63 × 10^–10^
		Group1: Cluster5 *vs.* Group2: Rest all clusters	1.81 × 10^–01^
		Group1: Cluster9 *vs.* Group2: Rest all clusters	1.19, ×, 10^–03^
*Tkt*	$\frac{4}{10}$	Group1: Cluster1 *vs.* Group2: Rest all clusters	1.24 × 10^–05^
		Group1: Cluster2 *vs.* Group2: Rest all clusters	5.81 × 10^–05^
		Group1: Cluster6 *vs.* Group2: Rest all clusters	1.68 × 10^–02^
		Group1: Cluster7 *vs.* Group2: Rest all clusters	2.44 × 10^–01^
*Tm*9*sf*3	$\frac{4}{10}$	Group1: Cluster0 *vs.* Group2: Rest all clusters	1.42 × 10^–03^
		Group1: Cluster4 *vs.* Group2: Rest all clusters	2.90, ×, 10^–14^
		Group1: Cluster5 *vs.* Group2: Rest all clusters	1.98 × 10^–02^
		Group1: Cluster9 *vs.* Group2: Rest all clusters	1.77 × 10^–03^
*Ywhae*	$\frac{4}{10}$	Group1: Cluster0 *vs.* Group2: Rest all clusters	3.64 × 10^–09^
		Group1: Cluster3 *vs.* Group2: Rest all clusters	3.75 × 10^–08^
		Group1: Cluster5 *vs.* Group2: Rest all clusters	9.12 × 10^–05^
		Group1: Cluster9 *vs.* Group2: Rest all clusters	2.26 × 10^–02^

a See Supplementary Table S2 for details.

**FIGURE 9:**
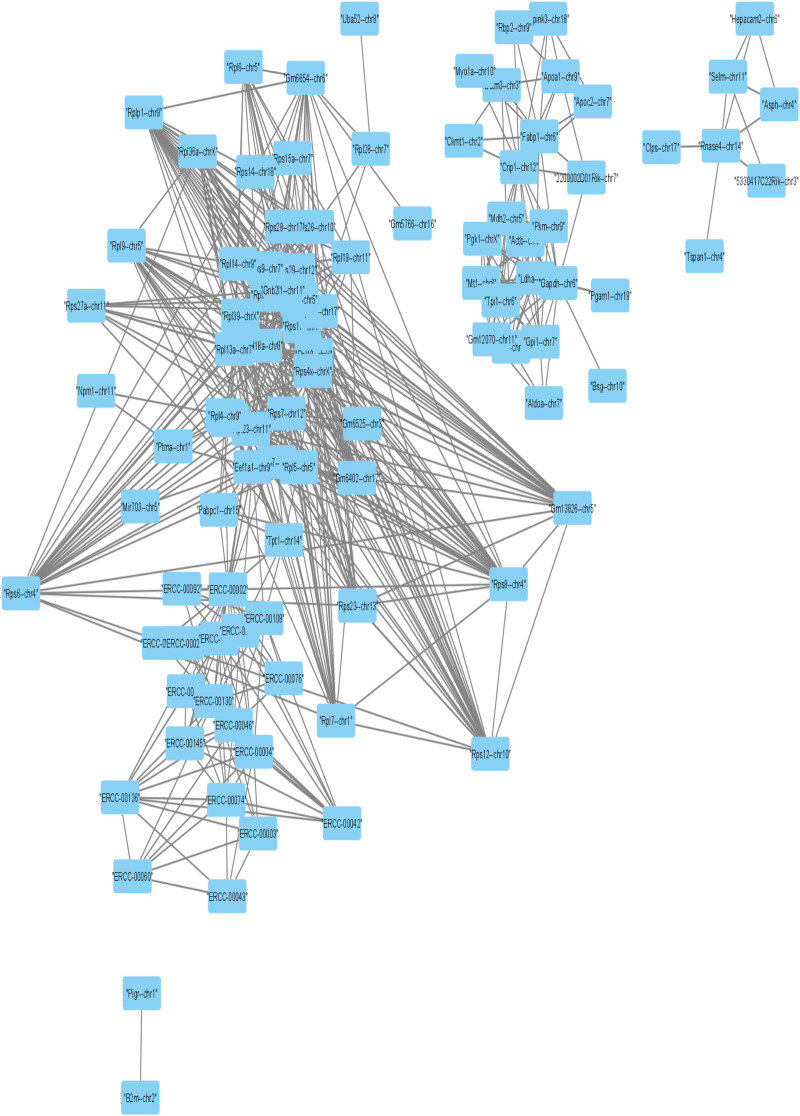
Visualization of Hub gene network of strongly correlated frequent markers.

Further, we applied Spearman’s correlation analysis on our cluster-specific most frequent markers or overlapped markers to detect edges among genes having correlation value greater than or equal to 0.5 (highly positively correlated) or, less than or equal to ( − 0.5) (highly negatively correlated). Then, we performed degree centrality hub gene network analysis using *Cytoscape* (Shannon et al., 2003). In our analysis, five genes with the highest degree values were *Rps4x*, *Rps18*, *Rpl13a*, *Rps12* and *Rpl18a*, see Table 2. We illustrated a detailed hub gene network structure in Figure 9.

**TABLE 2:** Top 20 hub genes ranked by degree centrality.

Gene symbol	Degree	Average shortest path length	Betweenness centrality	Closeness centrality	Clustering coefficient
*Rps4x*	32	1.800	0.056	0.556	0.536
*Rps18*	32	1.861	0.065	0.537	0.566
*Rpl13a*	31	1.877	0.021	0.533	0.596
*Rps12*	29	1.892	0.018	0.528	0.640
*Rpl18a*	29	1.923	0.012	0.520	0.662
*Gnb2l1*	29	1.923	0.036	0.520	0.589
*Rps8*	28	1.862	0.034	0.537	0.600
*Rps7*	28	1.661	0.099	0.602	0.587
*Rpl23*	28	1.646	0.170	0.607	0.582
*Rpl39*	27	1.877	0.032	0.533	0.587
*Rps17*	25	1.985	0.008	0.504	0.740
*Rps6*	24	1.923	0.018	0.520	0.677
*Rps9*	24	2.000	0.011	0.500	0.688
*Rpl3*	23	2.015	0.005	0.496	0.794
*Rpl7*	23	1.969	0.010	0.508	0.735
*Eef1a1*	22	1.785	0.142	0.560	0.420
*Gm13826*	22	2.046	0.004	0.489	0.770
*Rplp1*	22	2.323	0.007	0.430	0.675
*Gm6402*	21	2.000	0.018	0.500	0.628
*Rplp0*	21	2.338	0.008	0.428	0.652

In the corresponding literature survey, most of the top hub genes detected by our method played an important role of highly expressed markers or transcripts in exceptional nature of cell detection. *Rps4x* marker was considered as a highly expressed transcript in the study of An et al. (2014). and also played an important role to detect exceptional nature of the X chromosome by (Balaton et al., 2018). identified (Matarin et al., 2015) *Rps18* as the variant for minimizing the pairwise variation in gene expression through the hippocampal tissues from various mice. *Rpl13a* marker was found as housekeeping gene which is highly expressed in all types of cells by Wright et al. (2019) and also was identified as an up-regulated marker to recover the rare CD 34 + cells in the study of Fa et al. (2021). The marker *Rps12* was found in the study of Wisdom et al. (2020) as the antibody increased expressed proliferation genes. Basak et al. (2018) established *Rpl18a* as a cluster-specific overlapping marker which lies in three clusters.

Furthermore, we performed Gene Set Enrichment Analysis with David 6.8 software using our 3871 cluster-specific markers (Dennis et al., 2003). We applied DAVID database on our cluster-specific markes to obtain all KEGG pathways and Gene Ontology (GO) terms [Biological Process (BP), Cellular Component (CC) and Molecular Function (MF)], accompanied by number of genes in that pathway or GO-term, enriched Bonferroni corrected p-value and FDR. We import our input dataset in the prescribed format of DAVID 6.8 software, i.e., list of gene name in one column, select *OFFICIAL*_*GENE*_*SYMBOL* as Identifier, select *Mus musculus* as Species. Significant pathways and GO-terms were described in below and more details are provided in Tables 3–6 *mmu03010:Ribosome* had a top significant KEGG pathway which has minimum FDR value (5.17 × 10^–40^). A total of 112 genes were associated in this pathway with enriched Bonferroni corrected p-value 7.54 × 10^–40^. Table 3 contains rest of the top ten significant KEGG pathways. We provided a list of all KEGG pathways in a Supplementary Table S3. Similarly, *GO:0006 412 translation* was one of the top significant GO-BP terms with FDR value 1.02 × 10^–39^. 195 genes were associated with this GO-BP term having enriched corrected p-value 1.04 × 10^–39^. Table 4 contains the remaining terms. We provided the list of all GO-BP terms in a Supplementary Table S4. Furthermore, we found *GO:0070 062 extracellular exosome* as one of the top significant GO-CC terms with FDR value 9.37 × 10^–172^. A total of 1045 genes were associated with this GO-CC term having enriched corrected p-value 1.09, ×, 10^–171^. The rest of the terms are shown in Table 5. We provided the list of all GO-CC terms in a Supplementary Table S5. Lastly, *GO:0044 822 poly(A) RNA binding* was one of the top significant GO-MF terms with minimum FDR value 2.82 × 10^–120^. A total of 538 genes were associated with this GO-MF term having the enriched corrected p-value 2.94 × 10^–120^. For details, see Table 6. We provided the list of all GO-MF terms in a Supplementary Table S6.

**TABLE 3:** Top significant KEGG Pathways (FDR sorted).

KEGG pathway name^a^	#genes	Enriched Adjusted p-value	FDR
*mmu03010:Ribosome*	112	7.54 × 10^–40^	5.17 × 10^–40^
*mmu01100:Metabolic pathways*	477	3.97 × 10^–33^	1.36 × 10^–33^
*mmu01130:Biosynthesis of antibiotics*	124	5.34 × 10^–25^	1.22 × 10^–25^
*mmu00190:Oxidative phosphorylation*	89	3.77 × 10^–22^	2.58 × 10^–22^
*mmu05016:Huntington’s disease*	111	1.50, ×, 10^–20^	2.05 × 10^–21^
*mmu05012:Parkinson’s disease*	90	2.79, ×, 10^–19^	3.19, ×, 10^–20^
*mmu05010:Alzheimer’s disease*	100	1.19, ×, 10^–18^	1.16 × 10^–19^
*mmu04932:Non-alcoholic fatty liver disease (NAFLD)*	86	1.14 × 10^–14^	9.74 × 10^–16^
*mmu01200:Carbon metabolism*	70	1.50, ×, 10^–14^	1.14 × 10^–15^
*mmu03040:Spliceosome*	74	9.63 × 10^–13^	6.57 × 10^–14^

a See Supplementary Table S3 for details.

**TABLE 4:** Top significant GO-BP term enriched (FDR sorted).

GO-BP term name^a^	#genes	Enriched Adjusted p-value	FDR
*GO:0006 412 translation*	195	1.04 × 10^–39^	1.02 × 10^–39^
*GO:0006 810 transport*	529	6.16 × 10^–28^	3.02 × 10^–28^
*GO:0055 114 oxidation-reduction process*	241	2.04 × 10^–23^	6.69 × 10^–24^
*GO:0098 609 cell-cell adhesion*	93	1.04 × 10^–17^	2.55 × 10^–18^
*GO:0015 031 protein transport*	198	4.07 × 10^–15^	7.99, ×, 10^–16^
*GO:0006 397 mRNA processing*	127	6.55 × 10^–15^	9.33 × 10^–16^
*GO:0008 380 RNA splicing*	104	6.66 × 10^–15^	9.33 × 10^–16^
*GO:0016 192 vesicle-mediated transport*	93	2.03 × 10^–13^	2.48 × 10^–14^
*GO:0006 629 lipid metabolic process*	155	2.15 × 10^–11^	2.17 × 10^–12^
*GO:0008 152 metabolic process*	156	2.22 × 10^–11^	2.17 × 10^–12^

a See Supplementary Table S4 for details.

**TABLE 5:** Top significant GO-CC term enriched (FDR sorted).

GO-CC term name^a^	#genes	Enriched Adjusted p-value	FDR
*GO:0070 062 extracellular exosome*	1045	1.09, ×, 10^–171^	9.37 × 10^–172^
*GO:0005 739 mitochondrion*	632	6.43 × 10^–82^	2.76 × 10^–82^
*GO:0030 529 intracellular ribonucleoprotein complex*	209	7.92 × 10^–76^	2.26 × 10^–76^
*GO:0005 737 cytoplasm*	1669	1.38 × 10^–74^	2.96 × 10^–75^
*GO:0005 829 cytosol*	609	2.06 × 10^–64^	3.53 × 10^–65^
*GO:0005 840 ribosome*	131	2.87 × 10^–51^	4.10, ×, 10^–52^
*GO:0016 020 membrane*	1646	9.62 × 10^–49^	1.18 × 10^–49^
*GO:0005 634 nucleus*	1453	2.65 × 10^–47^	2.84 × 10^–48^
*GO:0005 654 nucleoplasm*	590	2.30, ×, 10^–43^	2.19, ×, 10^–44^
*GO:0005 743 mitochondrial inner membrane*	192	2.92 × 10^–43^	2.50, ×, 10^–44^

a See Supplementary Table S5 for details.

**TABLE 6:** Top significant GO-MF term enriched (FDR sorted).

GO-MF term name^a^	#genes	Enriched Adjusted p-value	FDR
*GO:0044 822 poly(A) RNA binding*	538	2.94 × 10^–120^	2.82 × 10^–120^
*GO:0098 641 cadherin binding involved in cell-cell adhesion*	158	6.77 × 10^–43^	3.25 × 10^–43^
*GO:0005 515 protein binding*	1046	1.15 × 10^–36^	3.68 × 10^–37^
*GO:0003 735 structural constituent of ribosome*	137	2.83 × 10^–31^	6.29 × 10^–32^
*GO:0003 723 RNA binding*	285	3.28 × 10^–31^	6.29 × 10^–32^
*GO:0000 166 nucleotide binding*	551	8.93 × 10^–28^	1.43 × 10^–28^
*GO:0019 899 enzyme binding*	143	3.90 × 10^–15^	5.34 × 10^–16^
*GO:0016 491 oxidoreductase activity*	196	5.39 × 10^–14^	6.46 × 10^–15^
*GO:0032 403 protein complex binding*	131	7.38 × 10^–13^	8.61 × 10^–14^
*GO:0019 904 protein domain specific binding*	111	4.18 × 10^–12^	3.95 × 10^–13^

a See Supplementary Table S6 for details.

## 4 Conclusion and Future Work

In this article, we provided a framework using dimensional reduction and cell clustering for identifying cluster-specific frequent biomarkers in single-cell sequencing data. To develop the framework, we first filtered our single-cell RNA sequencing dataset by discarding the features with a few numbers of cells and the cells with a few numbers of features. Then, we stored data and the result of analysis as a Seurat object and conducted many steps of the analysis such as using quality control metrics for cells filtration, discarding low quality or dying cells, computing cell-to-cell highly variable features from the dataset, and applying linear transformation and linear dimensionality reduction technique, PCA to project high dimensional data to an optimal low-dimensional space. We identified fifty “significant” principal components (PCs) based on strong enrichment of low p-value features and applied graph based clustering, modularity optimization agglomerative clustering algorithm, Louvain, on the cell of first 10 PCs and got 10 clusters with maximum modularity 0.885. Then we identified 3871 cluster specified biomarkers using downstream analysis through statistical test “MAST” by considering only up regulated differentially expressed genes (DEGs) as cluster marker with   log _2_ *FC* threshold 0.25 and minimum percentage of feature detection 25*%*. From these cluster specified biomarkers, we found 1892 most frequent markers, i.e., overlapping biomarkers.

Afterwards, we performed degree hub gene network analysis using Cytoscape (Shannon et al., 2003) and reported the five highest degree genes (*Rps4x*, *Rps18*, *Rpl13a*, *Rps12* and *Rpl18a*). Interestingly, our top hub genes are mainly composed of ribosomal protein genes. The biological explanation of ribosomal protein genes in the top hub genes are in single cell analysis, ribosomal protein genes are the most highly expressed genes in most cell types. Ribosomal protein genes play an efficient role for cell growth and proliferation (Petibon et al., 2021). Furthermore, we used pathway analysis on cluster specified markers using David 6.8 software (Dennis et al., 2003). In conclusion, our framework is useful for biological interpretation of the single-cell sequencing data analysis and efficiently identifying the cluster-specific overlapping biomarkers. As an advantage of our work, we can mention that due to the growing field of single cell sequencing analysis, some new approaches are encountered recently. Every technology has different strengths and weaknesses, and measurements are only based on some particular aspects of cellular identity, motivating the need to leverage information in one dataset to improve the interpretation of another. As an example, single cell ATAC-seq (scATAC-seq) can uniquely reveal enhancer regions and regulatory logic, but it is not possible to currently achieve the same power for unsupervised cell type discovery as transcriptomics (Lake et al., 2018). In other hand, STARmap method enables the measurement of more than 1,000 genes in spatially intact tissue, however forecast this number of genes as an upper limit for such approaches without super-resolution microscopy or the physical expansion of hydrogels (Wang et al., 2018). In our framework, we have tried to develop effective tools for single cell datasets which can enable similarly transformative advances in our ability to analyze and interpret single cell data.

The shortcoming of our framework is, here we have used raw data matrix in the place of imputed matrix. In our future study, we will improve our framework by using imputed data matrix. Besides, we will extend our current work by importing multi-objective optimization technique in clustering procedure to obtain a better clustering result. It can be applied on big data analysis, rare cell detection in single-cell RNA sequencing data analysis.

## Data Availability

Publicly available datasets were analyzed in this study. This data can be found here: https://www.ncbi.nlm.nih.gov/geo/, GSE62270. The code employed for this study can be found here: https://github.com/soumita-seth/single-cell-rna-sequencing-code.git.
