# Supplementary figures and images for: Dimensionality Reduction and Louvain Agglomerative Hierarchical Clustering for Cluster-Specified Frequent Biomarker Discovery in Single-Cell Sequencing Data
Source: Front Genet. 2022 Feb 7;13:828479. doi: 10.3389/fgene.2022.828479 (PMC8859265; doi:10.3389/fgene.2022.828479)

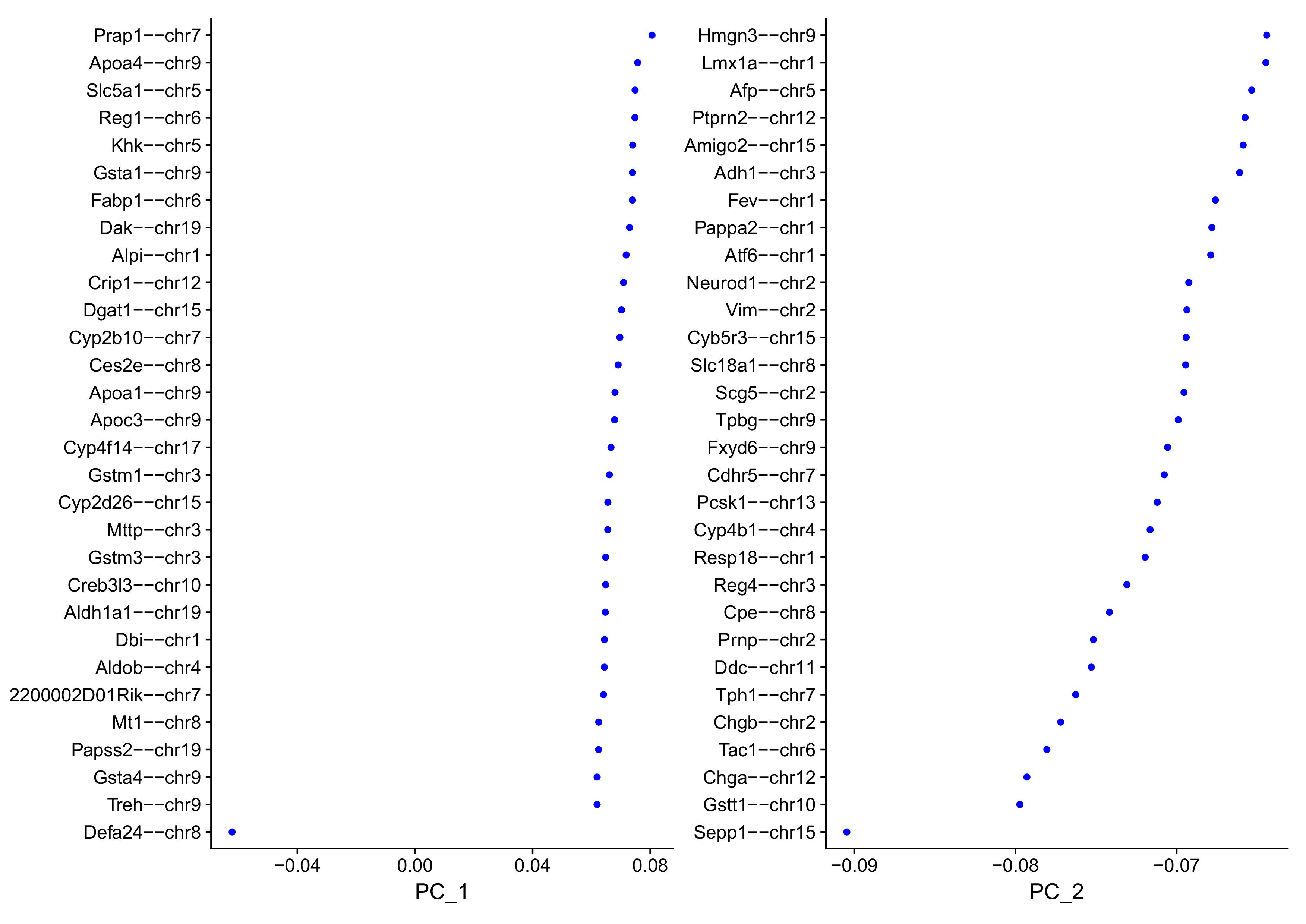

Supplement: Supplementary file 3 [file Image1.JPEG]
